# Infectious microbial diseases and host defense responses in Sydney rock oysters

**DOI:** 10.3389/fmicb.2014.00135

**Published:** 2014-04-23

**Authors:** David A. Raftos, Rhiannon Kuchel, Saleem Aladaileh, Daniel Butt

**Affiliations:** ^1^Department of Biological Sciences, Macquarie UniversityNorth Ryde, NSW, Australia; ^2^Sydney Institute of Marine ScienceMosman, NSW, Australia; ^3^Electron Microscope Unit, University of New South WalesKensington, NSW, Australia; ^4^Department of Biology, Al - Hussein Bin Talal UniversityAmman, Jordan

**Keywords:** oysters, disease, aquaculture, selective breeding, environmental stress

## Abstract

Aquaculture has long been seen as a sustainable solution to some of the world's growing food shortages. However, experience over the past 50 years indicates that infectious diseases caused by viruses, bacteria, and eukaryotes limit the productivity of aquaculture. In extreme cases, these types of infectious agents threaten the viability of entire aquaculture industries. This article describes the threats from infectious diseases in aquaculture and then focuses on one example (QX disease in Sydney rock oysters) as a case study. QX appears to be typical of many emerging diseases in aquaculture, particularly because environmental factors seem to play a crucial role in disease outbreaks. Evidence is presented that modulation of a generic subcellular stress response pathway in oysters is responsible for both resistance and susceptibility to infectious microbes. Understanding and being able to manipulate this pathway may be the key to sustainable aquaculture.

## Introduction

**Figure 1:**
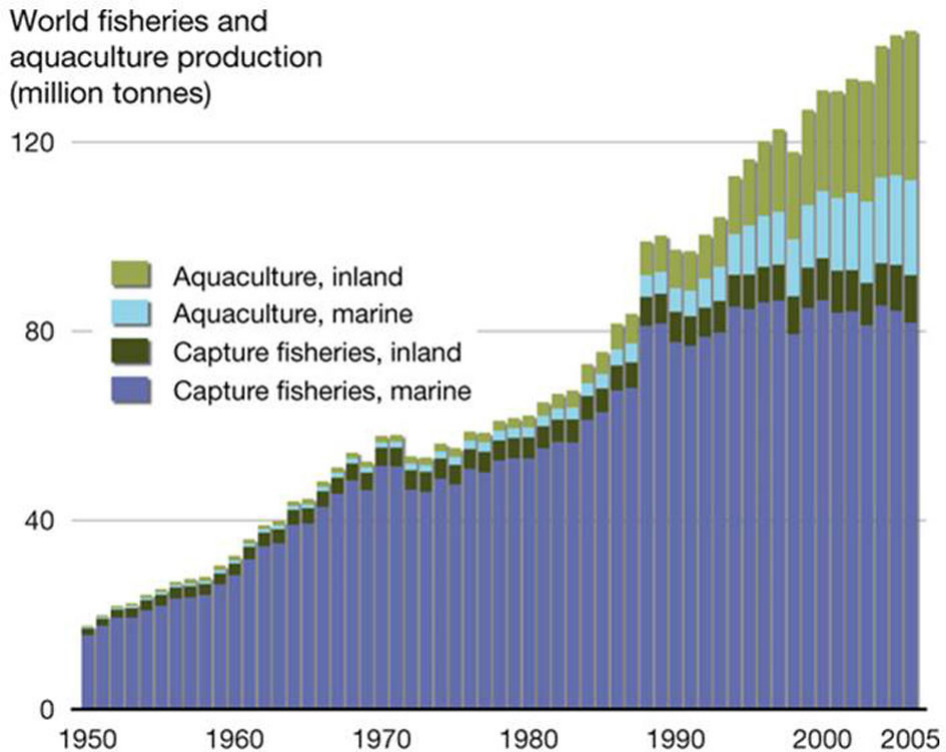
**World aquaculture production between 1980 and 2010, showing the contribution from different growing environments (from: http://2011.polarhusky.com/logistics/maps-and-route/data/world-fisheries/)**.

During the latter part of the last century, aquaculture (the farming of aquatic animals and plants) was seen as a key emergent food source needed to compensate for the world's rapidly growing human population. Aquaculture production increased rapidly during the 1970's and 1980's (FAO, 2012) (Figure 1). However, despite continued strong growth in freshwater aquaculture (primarily in Asia), increases in production from farming of marine species, such as oysters, has slowed. In some cases, output has begun to decline. There are a number of reasons for this. Marine aquaculture (mariculture) is susceptible to environmental degradation of coastal and estuarine waters around the world, and the supply of new, accessible farming sites is limited. More importantly, mariculture is particularly prone to infectious diseases, which are the main limiting factors in marine aquaculture production worldwide (Leung and Bates, 2013).

The pathogens and parasites that affect aquaculture production around the world include viruses [e.g., ostreid herpes virus (OsHV1), white spot syndrome virus (WSSV), abalone viral ganglioneuritis], bacteria (e.g., *Vibrio harveyi*, *Flexibacter columnaris*, *Aeromonas salmonicida*), protozoans (e.g., *Perkinsus* species, *Marteilia* species, and *Bonamia* species), and multicellular parasites or pests (e.g., mud worms and platyhelminths) (Renault, 1995; Coelen, 1997). These infectious agents can be highly host specific (e.g., *Marteilia sydneyi* in Sydney rock oysters) or have a broad range of host species (e.g., *Perkinsus olseni*, WSSV, or *Vibrio harveyi*). Disease epizootics caused by these infectious agents are often devastating. For instance, outbreaks of WSSV are responsible for annual losses in the shrimp industry of up to $10 billion worldwide (Flegel and Alday-Sanz, 2007; Sánchez-Martínez et al., 2007; FAO, 2012), whilst OsHV1 microvariant (OsHV-1 μvar) epizootics between 2008 and 2012 caused the production of Pacific oysters (*Crassostrea gigas*) in France to decline by 40% (Segarra et al., 2010).

Factors that affect the susceptibility of mariculture industries to disease epizootics include zoonoses altering the host specificity of pathogens and the ingression of pathogens from wild populations, reliance on high density monocultures, ease of transmission in the aquatic environment, the availability of intermediate hosts, and environmental degradation as a result of human activities (McCallum et al., 2003; Martin et al., 2010; Pulkkinen et al., 2010). This article focuses on the effects of environmental stress on disease susceptibility in aquaculture. It uses a specific example, QX disease in Sydney rock oysters, to highlight the complex relationships that exist between pathogens, their hosts and the environment.

## The sydney rock oyster industry

The Sydney rock oyster, *Saccostrea glomerata* (previously known as *S. commercialis*), has been cultivated in Australian coastal waters since the 1870's. A decline in natural oyster stocks following European settlement led to the establishment of the first cultivation techniques in state of New South Wales (NSW) in 1872 (Nell, 1993). Initial oyster farming techniques were based on the Claire (ponds) method used throughout France. In the 1930's, the Sydney rock oysters industry adopted a new cultivation system, in which oyster larvae were caught on tarred hardwood sticks placed in estuaries where spatfall is reliable (Angell, 1986). Traditionally, sticks were moved around bays or estuaries after spatfall and oysters were grown to maturity in the inter-tidal zone. More recently, farmers implemented a “single seed” culture method, which involves spat being scraped off sticks after settlement. The juvenile oysters are then grown in trays or baskets in sub-tidal and inter-tidal zones (Nell, 1993). The single seed method for capturing spat is now being replaced by hatchery technologies, where spat are produced in hatchery-based selective breeding programs that can control the phenotypic characteristics of oysters.

**Figure 2:**
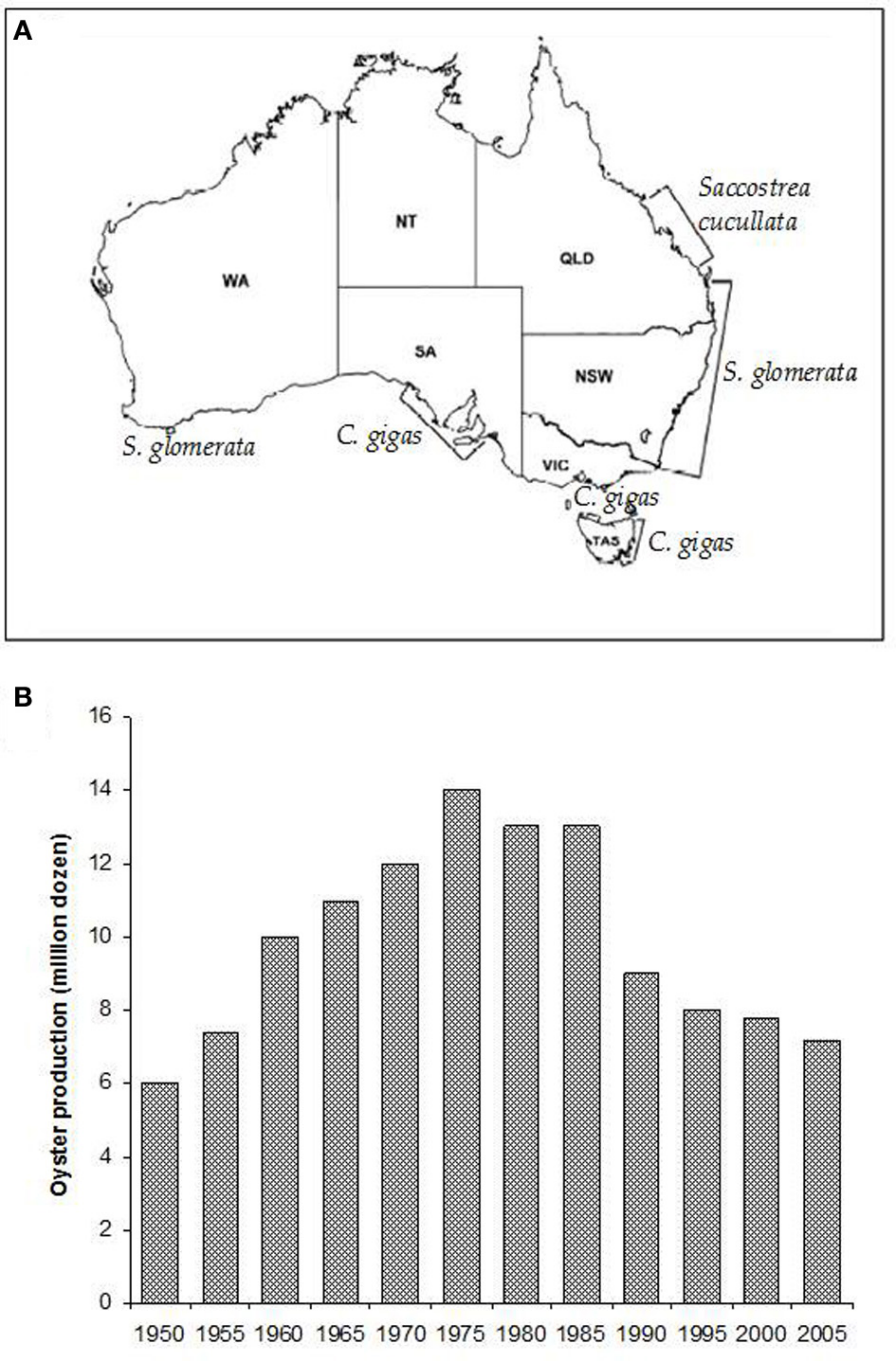
**(A)** The major oyster growing areas of Australia and the species farmed in those areas (Nell, 2003). **(B)** Sydney rock oyster production grew steadily until the mid-1970's, when pressure from infectious disease epizootics caused a major decline in the industry (Nell, 2003).

Sydney rock oysters are produced on the eastern Australian seaboard from the NSW/Victorian border in the south to Moreton Bay in subtropical Queensland (Nell, 1993) (Figure 2A). There has also been some success in farming the species on the north coast of Australia and in Western Australia (Nell, 2002).

Sydney rock oyster farming is the fourth biggest oyster aquaculture industry in the world and remains NSW's largest aquaculture industry. However, production levels have fallen by over 40% since the 1970's and remain fragile (Figure 2B; Heasman et al., 2000). Three thousand eight hundred and eighty three tonnes of edible oysters were produced in NSW in 2010–2011 (ABARE, 2012). This represents a 22% (1077 tonne, $4.7 million) decrease in edible oyster production compared with 2009–2010. The substantial decline in Sydney rock oyster production since its peak in the 1970's has been due primarily to the impacts of two infectious diseases, Winter Mortality Syndrome, and QX disease.

## Oyster diseases

Since the mid-1800's, when overfishing and destruction of natural oyster beds led to the development of oyster farming throughout Europe, disease has been the prominent controlling factor in oyster population dynamics (Roch, 1999). Global oyster production is based almost entirely upon just five species. Hence, oyster industries are usually local monocultures that are subject to inherent disease epizootics. Apart from OsHV1 in Pacific oysters, the majority of oyster diseases are caused by protozoan parasites. The estuarine environments where oysters are cultured provide an ideal medium for the dispersal and survival of parasitic protozoans due to natural currents, stable temperatures, and availability of intermediate hosts. The two main protozoan diseases affecting production of Sydney rock oysters (Winter Mortality and QX disease) only infect *S. glomerata*. The transfer of oysters between estuaries for on-growing during the 1960's was originally thought to have aided the spread of both diseases (Nell, 2003).

The main etiological agent of Winter Mortality is a protist *Bonamia roughleyi* (previously known as *Mikrocytos roughleyi;* Cochennec-Laureau et al., 2003). This includes the parasite in a genus that also commonly afflicts European flat oysters. As its name suggests, Winter Mortality predominantly occurs during colder months from June to August and is restricted to the cooler southern range of Sydney rock oysters. In affected areas, mortalities of up to 80% are common. Oysters in their third winter, just prior to reaching market size, are the most susceptible (Smith et al., 2000). Current methods used to manage the disease include transferring oysters to upstream leases where lower salinities are thought to decrease susceptibility to *B. roughleyi* infection. Many farmers also sell their oysters prior to their third winter when they are most at risk of infection. However, this option is rarely available to growers in the more southerly regions, because oysters in these colder waters take longer to reach market size (Smith et al., 2000). New information also suggests that other pathogens, in addition to *B. roughleyi*, may be involved in Winter Mortality.

## *Marteilia sydneyi* and QX disease

**Figure 3:**
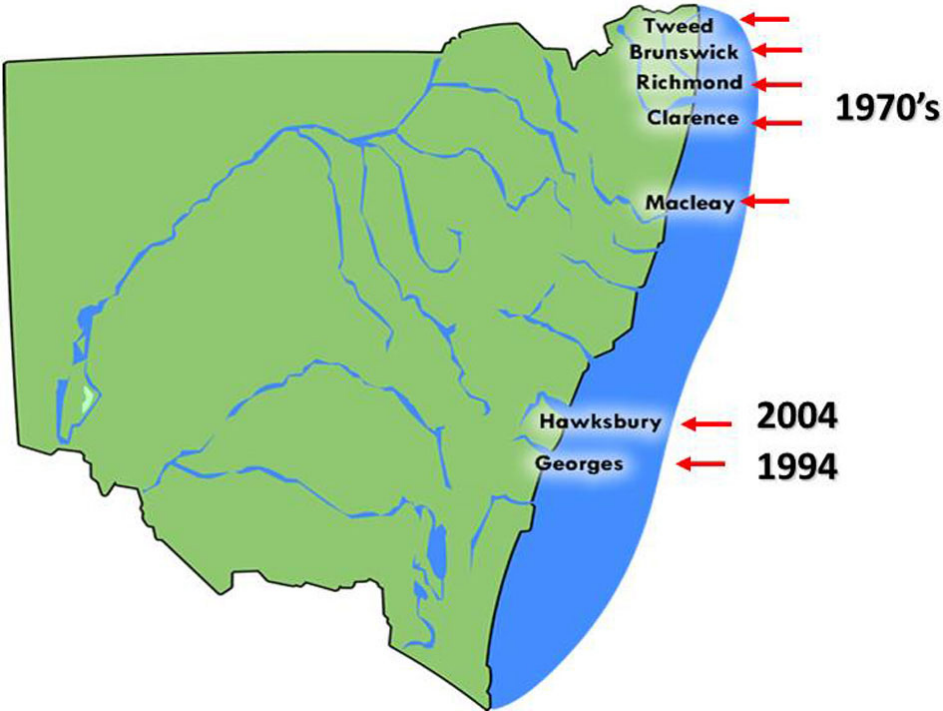
**The distribution of Sydney rock oyster farming estuaries on the coast of NSW, Australia that are affected by QX disease and the year in which QX disease outbreaks first occurred**.

QX (for Queensland Unknown) disease, also known as marteiliosis, is now the most serious disease affecting the Sydney rock oyster industry (Anderson et al., 1995; Adlard and Ogburn, 2003). QX disease is caused by the paramyxean protozoan *Marteilia sydneyi*, which was first described by Perkins and Wolf (1976). The infective season for *M. sydneyi* occurs during the southern hemisphere summer and early autumn, from January to April. Outbreaks of QX disease were first recorded during the late 1970's and were restricted to a small number of estuaries from The Great Sandy Straight of southern Queensland through to the Macleay River in northern NSW (Adlard and Ernst, 1995) (Figure 3).

The parasite was later discovered in the Georges River, Sydney, in 1994. Examination of *S. glomerata* digestive tract samples taken from Lime Kiln Bar (33°59′08″S, 151°03′10″E) in the Georges River identified tricellular sporonts that were typical of *M. sydneyi* infections (Adlard and Ernst, 1995). During 1993/1994 over 13 million oysters were produced in the Georges River. However, consistent seasonal outbreaks of QX disease have caused production to steadily decline so that only 750,000 oysters were produced there in 2000/2001. This constitutes a fall in production of 94% over 6 years (Nell and Hand, 2003). Mortality rates from QX disease can reach 95% per year (Peters and Raftos, 2003). Current production in the Georges River is restricted to selectively bred, *M. sydneyi*-resistant strains. Pacific oysters, which are unaffected by QX disease, were introduced as a replacement for Sydney rock oysters in the Georges River. However, these Pacific oysters have since been devastated by outbreaks for OsHV1 μvar, which began in 2010.

The Hawkesbury River, approximately 50 km north of the Georges River in NSW, is the most recent growing area to experience an outbreak of QX disease. This estuary had previously been the second largest producer of Sydney rock oysters in Australia. The disease was first detected in the Hawkesbury River during 2004, when an examination of oysters from upstream oyster leases uncovered sporulating *M. sydneyi* in oyster guts. By the end of 2005, Sydney rock oysters in most growing areas of the estuary had suffered mortality rates of up to 90% (Butt and Raftos, 2007). Again, Pacific oysters were used to replace Sydney rock oysters in the Hawkesbury River, and again they were devastated (up to 100% mortality) by an outbreak of OsHV1 μvar in 2013.

A sensitive, polymerase chain reaction (PCR) diagnostic assay for *M. sydneyi* has been used to test for the presence of the parasite in numerous estuaries along the NSW coastline (Adlard and Worthington-Wilmer, 2003). These tests have shown that *M. sydneyi* is present in the vast majority of *S. glomerata* growing estuaries on the NSW coast, including those that had previously been thought to be parasite free (Adlard and Wesche, 2005). There are approximately 40 estuaries in which Sydney rock oysters are grown, but only 7 of these have experienced QX disease outbreaks. Based on these data, it is now widely believed that *M. sydneyi* is an enzootic parasite of Sydney rock oysters throughout Australia (Adlard and Wesche, 2005).

## The *M. sydneyi* life cycle

**Figure 4:**
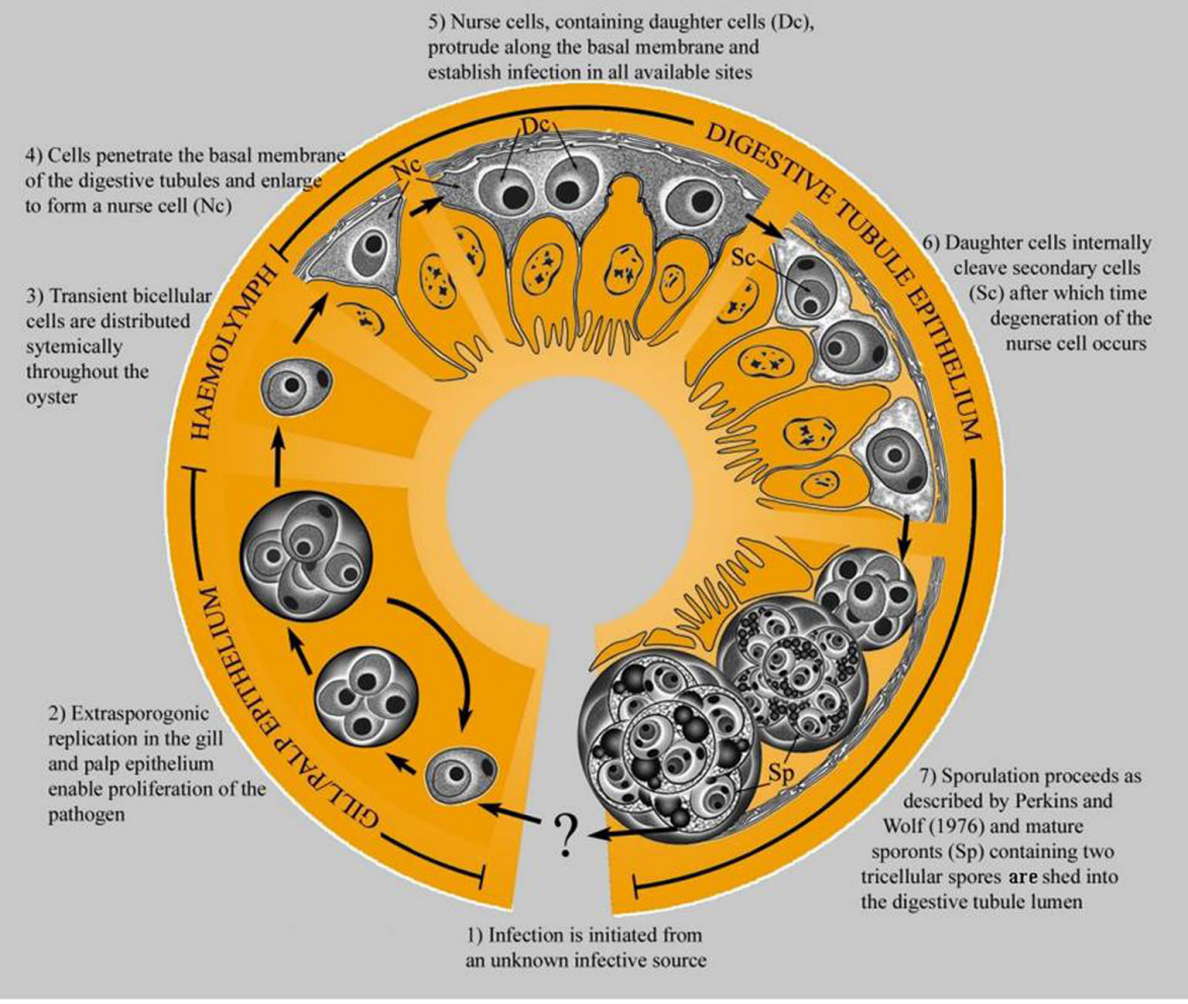
**The life cycle of *M. sydneyi* within Sydney rock oysters (Kleeman et al., 2002)**.

The original description of *M. sydneyi* by Perkins and Wolf (1976) focused on pathogen sporulation in the digestive gland of the oyster host. Traditional laboratory detection techniques limited further elucidation of *M. sydneyi's* early development. However, the use of DNA probes for *in situ* hybridization has allowed the site of initial infection and subsequent development of the parasite within oysters to be defined (Figure 4) (Kleeman et al., 2002). The earliest infectious stage of *M. sydneyi* that can be identified in oysters is a uninucleate stem cell. These stem cells were discovered in the palps and gill epithelia of *S. glomerata*. Their presence in these epithelial tissues suggests that infection results from a “free-floating” parasitic stage entering the gills during filter feeding (Kleeman et al., 2002). Stem cells proliferate in the gill epithelium. Once sufficient numbers of stem cells have been generated, they penetrate the basal membrane, entering connective tissues. This enables their dissemination throughout the oyster.

**Figure 5:**
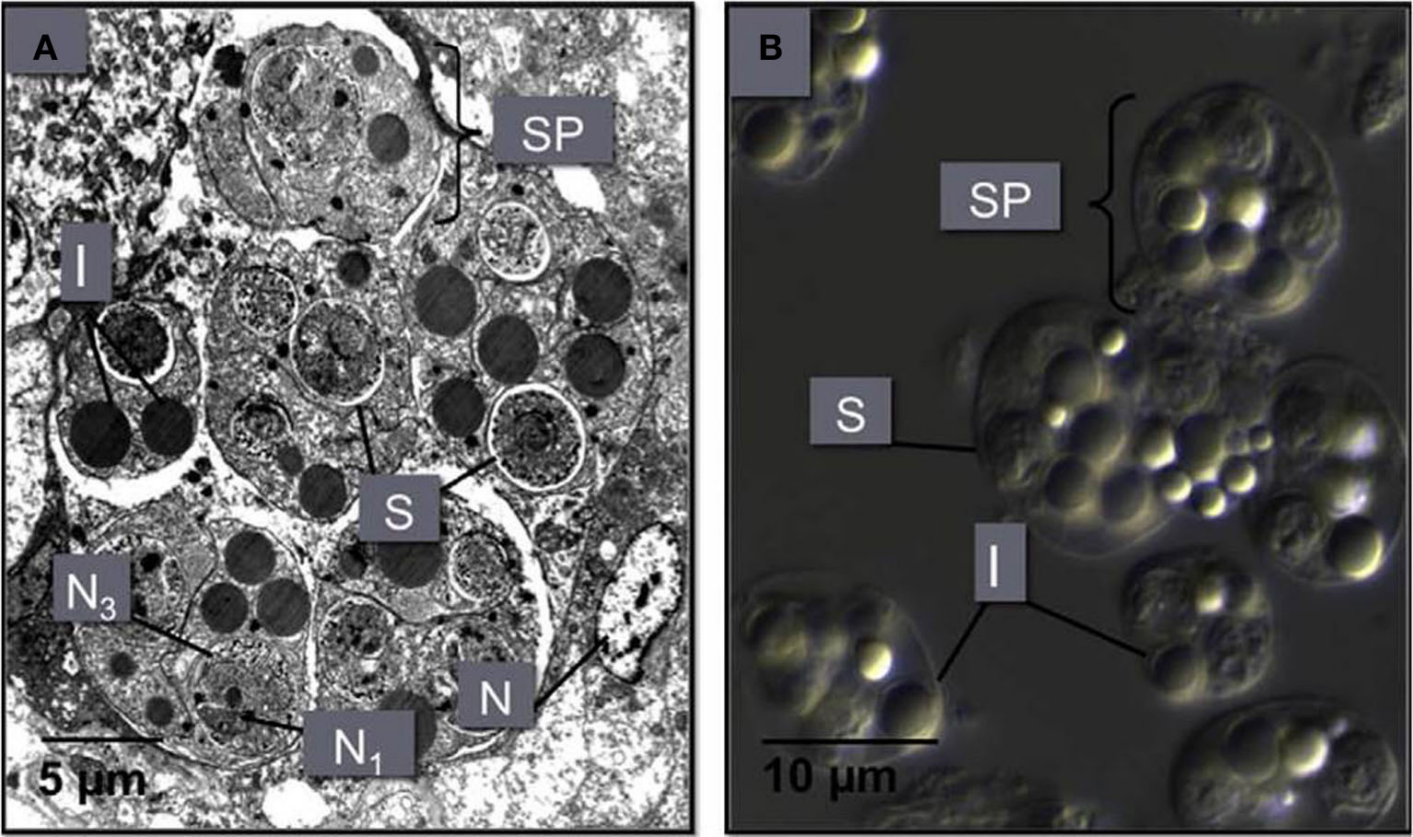
**(A)** An *M. sydneyi* sporangiosorus in the digestive gland of an infected oyster. This sporangiosorus contains six individual reproductive bodies (sporonts or secondary cells, SP). Cleavage of the sporangium (cytoplasm of the sporont) leads to the development of two to three multinucleated (N1, N3) spores (S), surrounded by inclusion bodies (I). The nucleus (N) of an oyster hemocyte adjacent to the sproangiosorus is also shown. **(B)** A differential interference contrast micrograph of *M. sydneyi* sporonts purified by density gradient centrifugation (Kuchel et al., 2010a,b).

The majority of stem cells converge on the digestive gland, where they enter the digestive tubule epithelium (Kleeman et al., 2002) (Figure 5). Once in the digestive gland, replication and further development continues to form a primary 2-celled plasmodium. This plasmodium divides to form between 8 and 16 sporonts. Each sporont then undergoes further internal division to form two spores, each with three concentric cells (Roubal et al., 1988). Spores are shed into the environment via the alimentary canal prior to the death of the oyster (Anderson et al., 1995).

Despite our understanding of *M. sydneyi's* development within oysters, it is only recently that the fate of the parasite has been determined once it is shed from infected oysters. Wesche et al. (1999) assessed spore survival after their release into the environment. They found that spores are relatively short-lived outside their oyster host (7–35 days) when compared to the 3–10 month infection cycle of the pathogen within oysters. Also, no morphological development of the parasite could be detected outside the host and energy reserves were deemed to be insufficient for prolonged survival (Wesche et al., 1999).

These results indicated that there may be an intermediate host for *M. sydneyi*. The existence of an intermediate host was supported by transmission trials on the closely related *Marteilia refringens*, agent of Aber Disease of flat oysters in Europe (Berthe et al., 1998). Healthy European flat oysters did not become infected when inoculated with live *M. refringens* cells, regardless of inoculum strength. Cohabitation trials under laboratory conditions also failed to transmit the parasite between oysters. It was only when uninfected oysters were placed in natural environments within the endemic range of the parasite that infections occurred (Audemard et al., 2001). Further research by Audemard et al. (2002) found that a copepod, *Paracartia grani*, became infected with *M. refringens* after 7 days cohabitation with infected oysters. The population dynamics of *P. grani* in *O. edulis* growing areas also match its potential role as an intermediate host for *M. refringens* (Audemard et al., 2004). However, the full life cycle of *M. refringens* has not been closed because re-infection (*P. grani* to *O. edulis* transmission) experiments have not been successful.

It was originally thought that the intermediate hosts of the QX parasite, *M. sydneyi*, might be scavenging carnivores that ingest spores as they feed on dead oyster tissue. However, the subsequent discovery that *M. sydneyi* spores are released prior to the death of *S. glomerata* suggested that benthic or filter-feeding organisms are more likely intermediate hosts (Roubal et al., 1988). This was further supported by the observation that shed spores have a negative buoyancy and so were likely to sink to the sediment below oyster racks (Wesche et al., 1999). In 2000, Kleeman and Adlard developed PCR and *in situ* hybridization assays to finally resolve the identification of an intermediate host for *M. sydneyi*. Their assays targeted the ribosomal DNA of *M. sydneyi* so that potential hosts could be tested for the presence of the parasite with high precision and specificity. However, definitive evidence for an intermediate host remains unavailable.

## Oyster immune responses against *M. sydneyi*

**Figure 6:**
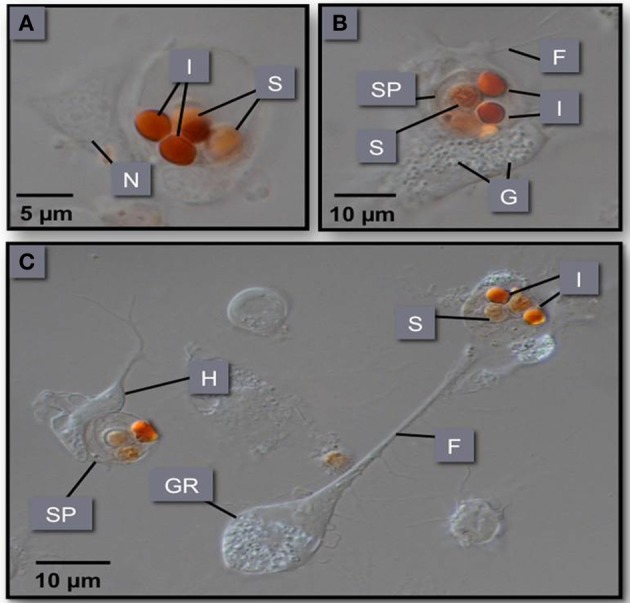
**(A–C)** Phagocytosis of *M. sydneyi* sporonts by *S. glomerata* hemocytes. H, hyalinocyte; GR, granulocyte; SP, sporont; I, inclusion bodies within sporonts stained with Congo red; F, filopodia; N, nucleus; G, intracellular granules within hemocytes.

Despite the lethality of *M. sydneyi* infections in some Sydney rock oyster growing areas, there is strong evidence to suggest that oysters can mount effective immune responses against *M. sydneyi*. Butt and Raftos (2007) examined the response of oyster hemocytes (the oyster equivalent of circulating blood cells) to *M. sydneyi in vitro*. They found that both granulocytes and hyalinocytes were able to rapidly ingest parasite sporonts by the process of phagocytosis (Figure 6).

**Figure 7:**
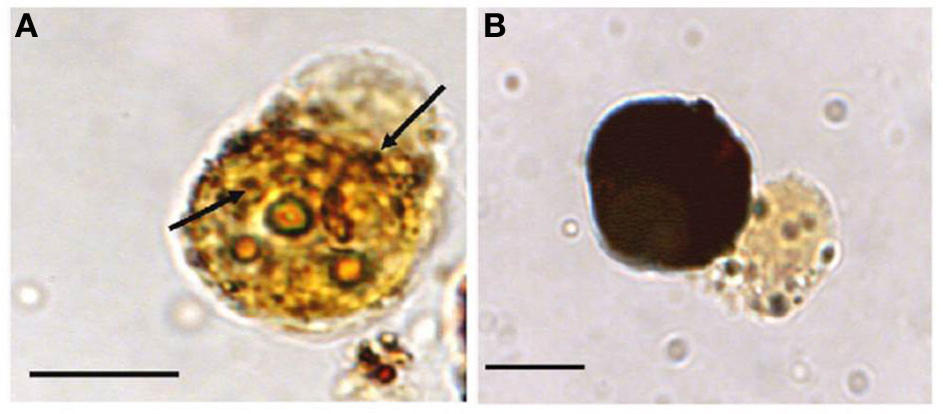
**Melanization of *M. sydneyi* by phenoloxidase activity within *S. glomerata* hemocytes. (A)** Initially, melanization (brown coloration) is focused around phagocytosed *M. sydneyi* sporonts. Arrows indicate granular melanin deposition around ingested parasite. **(B)** As melanization continues, entire hemocytes, including the internalized sporont(s), become melanized. Bars = 5 μm (Butt and Raftos, 2008).

**Figure 8:**
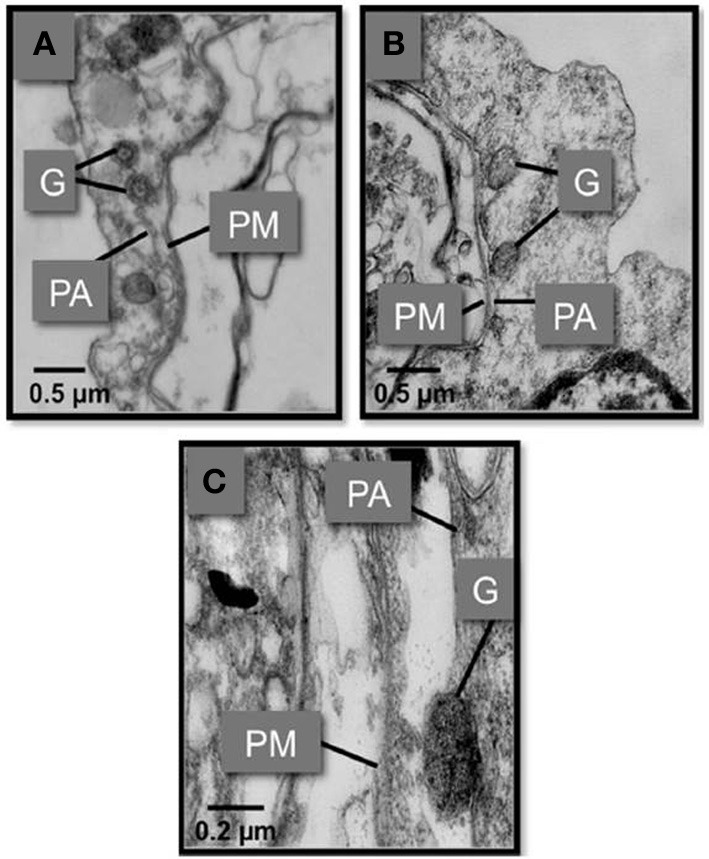
**Transmission electron micrographs showing the fusion of *S. glomarata* hemocyte granules with phagosomes containing ingested *M. sydneyi*. (A)** Hemocyte granules (G) approach and fuse to the phagosomal membrane (PA) that surrounds a *M. sydneyi* sporont. PM, parasite membrane. **(B)** Granules fuse to the phagosome containing *M. sydneyi* sporonts. **(C)** High resolution image of a granule fusing with the phagosome membrane around an *M. sydneyi* sporont (Kuchel et al., 2010a,b).

*In vitro* experiments showed that phagocytosis of *M. sydneyi* stimulated intracellular activity of the defensive enzyme, phenoloxidase. This led to the complete melanization of phagosomes containing parasites, and presumably the destruction of the ingested *M. sydneyi* (Figure 7). The role of phenoloxidase in phagolysosomal activity against *M. sydneyi* was supported by an electron microscopical study by Kuchel et al. (2010a). They showed that, after ingestion of *M. sydneyi*, granules in *S. glomerata* hemocytes that contain phenoloxidase (Aladaileh et al., 2007a), fuse with phagosome membranes and that the pH of phagosomes decreases in a typical phagolysosomal response (Figure 8).

In addition, Kuchel et al. (2010a) observed the deposition of phenoloxidase metabolites in phagosomes after *in vitro* phagocytosis of *M. sydneyi* sporonts by *S. glomerata* hemocytes. Most importantly, ingested and melanized *M. sydneyi* have also been detected *in vivo* among hemocytes from infected oysters (Butt and Raftos, 2008). All of these data suggest that Sydney rock oyster hemocytes can recognize and phagocytose *M. sydneyi*, and that phenoloxidase is a critical intracellular effector mechanism that acts against ingested *M. sydneyi* as part of the phagolysosomal process.

Phenoloxidase is a key enzyme in the immunological defenses of invertebrates (Söderhäll and Cerenius, 1998). The role of phenoloxidase in host defense is best characterized in arthropods, although numerous studies have also demonstrated its importance in bivalve molluscs (Deaton et al., 1999; Jordan and Deaton, 2005; Munoz et al., 2006; Aladaileh et al., 2007a; Hellio et al., 2007; Butt and Raftos, 2008). The enzyme facilitates the formation of the pigment melanin, which is important in the sequestration of foreign material during defensive encapsulation (Söderhäll and Cerenius, 1998). Melanin and its intermediate metabolites in the phenoloxidase pathway also have direct antimicrobial activities in both extracelullular fluids and intracellular phagolysosomes (Asokan et al., 1997).

We have made similar observations on the roles of phenoloxidase in the immune system of Sydney rock oysters. Aladaileh et al. (2007a) demonstrated that phenoloxidase is a key component of intracellular granules within *S. glomerata* phagocytes. It is also evident that intracellular phenoloxidase in *S. glomerata* can produce metabolites that are associated with antimicrobial activity (Aladaileh et al., 2007b), and that challenging Sydney rock oyster hemocytes with pathogen-associate molecules increases phenoloxidase activity (Aladaileh et al., 2007c).

## Environmental stress and disease in molluscs

Given that Sydney rock oysters clearly have effective immunological defenses against *M. sydneyi*, the outstanding question about QX disease is, why do oysters lose control of the parasite leading to outbreaks of lethal disease? Our work, described in detail below, suggests that environmental stress results in the suppression of the Sydney rock oyster immune system, and that this immunosuppression contributes to QX disease epizootics.

The survival of all organisms depends upon their ability to maintain homeostasis in highly variable environments. This balance is perhaps most difficult to maintain in sessile poikilothermic osmoconformers, such as bivalve molluscs. External stressors constantly threaten the physiological steady state of these organisms (Lacoste et al., 2002). Stressors to which oysters are exposed vary widely, but include extremes of temperature, salinity, and pH, as well as anthropogenic factors (Kuchel et al., 2011). The estuarine environment, which is home to commercial and wild oyster populations, is prone to extreme hydrological changes. These changes are associated with tidal fluctuations and rainfall events, and can lead to the introduction of effluent (nutrient loading and chemical contamination) and increased sediment loads resulting from upstream runoff. Oysters require a range of adaptive responses to counteract these stressors. Such responses, or the stressors themselves, often affect the effective function of physiological mechanisms, including the immune system. Immunosuppression, which can result from environmental stress, leaves organisms more susceptible to disease epizootics (Chu et al., 2002).

The link between aquaculture species, pathogens, and the environment has been acknowledged for some time (Kuchel et al., 2011). As long ago as 1974, it was recognized that disease outbreaks in fish only occurred when environmental conditions were suitable (Snieszko, 1974). However, mechanistic links between host immunological defense and environmental change has only been established far more recently. Lacoste et al. (2001a,b,c, 2002) have shown that oysters possess a form of catecholamine-based neuroendocrine response similar to the adrenergic system that is activated in vertebrates during acute stress responses. Their work demonstrated that, as environmental conditions change, immunological function in marine invertebrates can be inhibited by adaptive changes to their own physiology mediated by the catecholamine hormone, noradrenaline. Once released into the hemolymph, noradrenaline was shown to decrease hemocyte phagocytic activity and the production of reactive oxygen species (ROS) in phagolysosomes (Lacoste et al., 2001a,c).

Other studies have investigated the types of environmental or anthropogenic stressors that affect immunological activity in molluscs. Pipe et al. (1999) examined the effects of copper on various immunological parameters in the marine mussel, *Mytilus edulis*. They identified dose-dependent changes in hemocyte numbers, particularly a decrease in the frequency of circulating eosinophilic granulocytes, after copper exposure. These eosinophilic cells are responsible for most peroxidase, phenoloxidase, and phagocytic activity in *M. edulis* (Pipe et al., 1997). However, contrary to expectations, decreases in phenoloxidase and peroxidase activity in copper-affected mussels were not statistically significant. This could be explained in part by the large variability in the activity of these enzymes between individual mussels (Pipe et al., 1999).

Further work on the effects of anthropogenic pollutants on the *M. edulis* immune system showed that polycyclic aromatic hydrocarbons (PAH), such as fluoranthene and phenanthrene, inhibit phagocytic activity and damage lysosomes. PAH's accumulate within lysosomes so that damage is caused by direct physical disturbance of the lysosomal membrane. Similar disruption inhibits phagocytosis (Grundy et al., 1996). The combined stress of high temperature and copper exposure was also shown to have deleterious effects on *M. edulis* hemocytes. Both total and differential hemocyte counts were affected, as were superoxide production and phagocytic activities (Parry and Pipe, 2004).

Pacific oysters (*C. gigas*) have also been tested to determine the effects of mechanical disturbance on immunological function. The effects of mechanical agitation were investigated because oyster culture techniques require individuals to be continually sorted and redistributed throughout their development using mechanical grading machines. Lacoste et al. (2002) tested whether such disturbance made oysters more susceptible to disease outbreaks. They found that hemocyte chemotaxis, phagocytosis and ROS production were all inhibited after continuous shaking for 15 min. These immunological parameters rapidly recovered in the 60–90 min after shaking, indicative of an acute hormonal stress response. Similar results were reported when abalone (*Haliotus tuberculata*) were exposed to mechanical disturbance. Immediately after agitation, hemocyte numbers, as well as amoeboid, phagocytic, and superoxide activities, were significantly decreased. This was followed by a compensatory increase in most parameters 4 h after the stress (Malham et al., 2003). In both of these studies, noradrenaline and dopamine concentrations were measured as stress indicators. The concentrations of both catecholamine hormones increased immediately after the onset of the mechanical disturbance. Such results suggest a direct link between physical stress, hormonal responses, and immunological impairment (Lacoste et al., 2002; Malham et al., 2003).

However, other studies on oysters have demonstrated very different effects depending upon the type of stress applied. In Eastern oysters (*C. virginica*) exposure to various concentrations of tributyltin was found to have negligible effects on immunological activity (Anderson et al., 1996). Similarly, exposure to different salinity levels had no detectable effect on lysozyme or respiratory burst activities in the European flat oyster, *O. edulis* (Hauton et al., 2000). This contrasted studies looking at dietary effects on immunological activity in *C. gigas*. Zhang and Li (2006) found that starving oysters for 42 days reduced condition indices and lysosomal membrane integrity. Alternatively, improving nutrition in Pacific oysters increased oxidative activity and phagocytic clearance rates. These results were also replicated in similar trials using the Manila clam, *Ruditapes philippinarum* (Delaporte et al., 2003).

Normal physiological variables have also been shown to impair immunological activity. For instance, gametogenesis in Pacific oysters reduces phagocytic activity and hemocyte function (Delaporte et al., 2006). It is thought that these physiological changes associated with broadcast spawning could explain some of the seasonal variability in immunological activity observed in many molluscan species and their susceptibility to disease (Duchemin et al., 2007).

Even though the role of environmental stress in the impairment of immune responses has been established in a variety of aquaculture species, the effects that this immunological suppression has on disease susceptibility has only been investigated in a small number of host-pathogen relationships (Lafferty and Kuris, 1999). One study investigated the collapse of the Black abalone (*Haliotis cracherodii*) fishery on the Californian coast. Field studies concluded that the combination of numerous stressors, including increased water temperatures, pollutants, over-fishing, and competition from sea urchins left the abalone susceptible to the etiological agent of withered foot syndrome, *Xenohaliotis americanus* (Davis et al., 1992; Lafferty and Kuris, 1999). Laboratory studies using another abalone species, *Haliotis diversicolor supertexta*, found that exposures to ammonia, high temperatures and low dissolved oxygen concentrations all impaired cellular immunological responses. As a result, the abalone in all treatments showed increased susceptibility to infection by *Vibrio parahaemolyticus* (Cheng et al., 2004a,b,c).

Disease susceptibility in the Eastern oyster, *C. virginica*, has also been shown to increase in response to anthropogenic pollutants (Chu and Hale, 1994; Fisher et al., 1999). However, Chu et al. (2002) concluded that this increased susceptibility may not have been caused by immunological inhibition. Despite testing a range of cellular and humoral immunological parameters, no differences could be detected between control oysters and those exposed to contaminated sediments.

## The role of environmental stress in QX disease

Similar associations between environmental stress, the immune system and disease have been identified in QX disease outbreaks. Peters and Raftos (2003) found that inhibition of oyster immunological function (reflected by phenoloxidase activity) is associated with infective periods for *M. sydneyi*. The fact that phenoloxidase activity was suppressed in oysters that were not actively infected with *M. sydneyi* suggests that external influences, and not *M. sydneyi* itself, were responsible for this inhibition of the immune system.

Anecdotal evidence from oyster farmers had long pointed to a link between QX disease and environmental factors. Initial examination of QX disease in southern Queensland suggested that outbreaks occurred after heavy summer rainfall (Haysom, 1978; Lester, 1986). This prompted studies to determine whether epizootics of *M. sydneyi* were triggered by a drop in environmental pH associated with runoff from acid sulfate soils. Early work by Anderson et al. (1995) found that *M. sydneyi* infection still occurred during periods when no major pH fluctuations were observed. However, a subsequent study by Wesche (1995) found that infection outbreaks did occur soon after a major drop in environmental pH, even though no causal relationship between pH and QX disease outbreaks could be established. It was also demonstrated that substantial drops in pH could occur without resulting in a QX disease epizootic.

**Figure 9:**
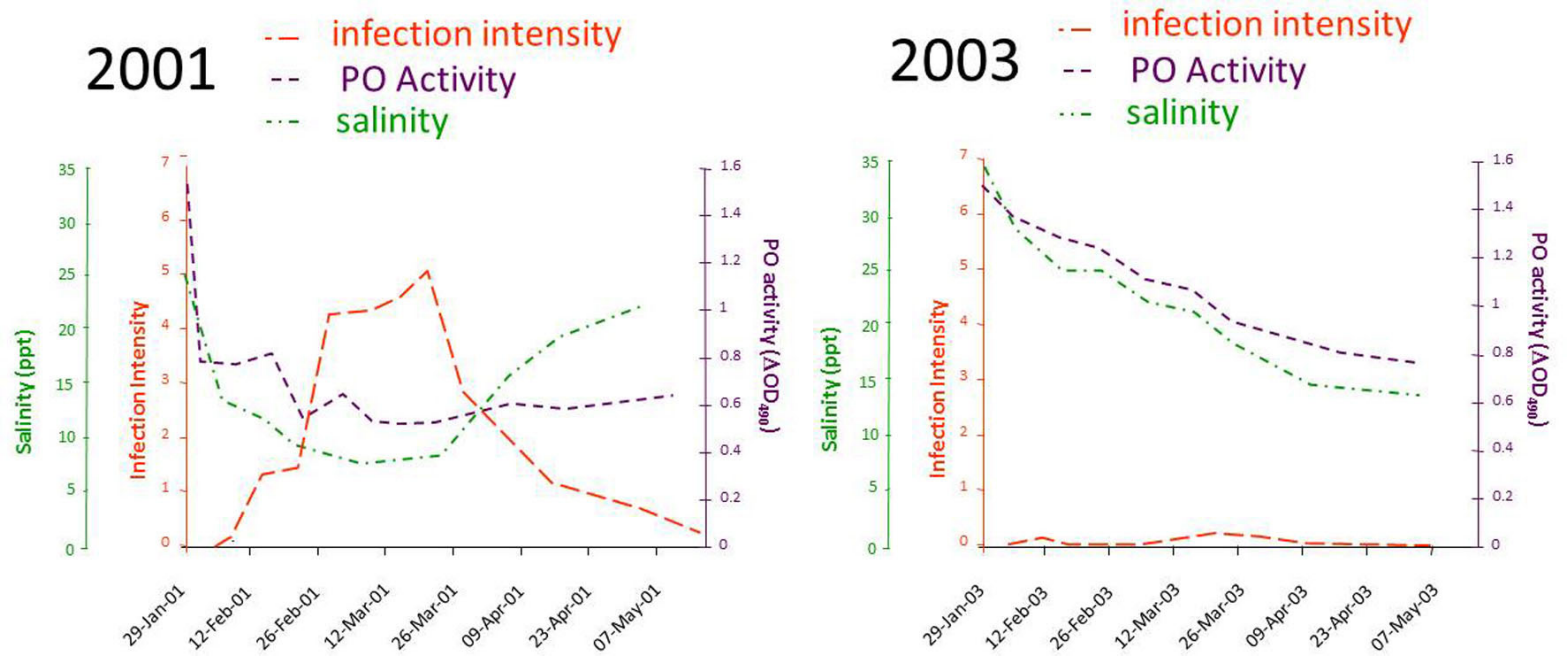
**Results of a field trial conducted in the Georges River, Sydney, between 2001 and 2004**. Data were recorded for the salinity of water in the river, the phenoloxidase (PO) activities in hemolymph from oysters held in the river, and the intensity of *M. sydneyi* infections within those oysters. Data were recalculated from those reported in Peters and Raftos (2003).

A breakthrough in linking QX disease to environmental stress came from a study by Peters and Raftos (2003), which again tested a relationship between rainfall and disease, this time focusing on low salinity rather than altered pH. They used field trials to show that the activity of the key defensive enzyme, phenoloxidase, was consistently lower in oysters held in QX disease prone areas, relative to those in QX disease free locations. This suggested that suppression of phenoloxidase activity may be responsible for QX disease outbreaks. Reanalysis of their data to include the salinity of the water at the different locations over time identified a strict relationship between low salinity associated with summer rainfall, suppression of phenoloxidase activity and increasing intensity of *M. sydneyi* infection. This work (Peters and Raftos, 2003) also provided an explanation for temporal variation in QX disease, wherein severe disease outbreaks can occur within an oyster growing area one year, but not the next. Their field studies in the Georges River showed substantial differences in salinity, phenoloxidase activity, and the intensity of *M. sydneyi* infection between 2001, a year in which seasonally average levels of rainfall were recorded in the Georges River catchment during summer, and 2003, which was the beginning of a major drought on the east coast of Australia (Figure 9).

The link between low salinity, immune suppression, and QX disease was later supported by Butt et al. (2006), who demonstrated that phenoloxidase activities were proportionally lower in oysters exposed under laboratory conditions to low (7 ppt) and intermediate (13.5 ppt) salinities, relative to those held under “normal” (34 ppt) conditions. This correlation between low salinity and low phenoloxidase activity was also evident in experiments in which oysters were held in water from different sites within the same river system that had different levels of salinity (Butt et al., 2006). Significantly, these data also matched historical observations that QX disease outbreaks are more severe in the low saline, upper reaches of estuaries. It may also explain why more estuaries are affected by QX disease in sub-tropical regions of Australia (northern NSW and southern Queensland) where average annual rainfall can be more than twice that of temperate (southern) areas.

Further investigation revealed that a range of environmental stressors, not just low salinity, have substantial effects on the Sydney rock oyster immune system that might be associated with disease susceptibility. For instance, Butt et al. (2008) showed that the muscle relaxant, magnesium chloride, which was being trialed for use in Sydney rock oyster hatcheries, significantly affected a range of parameters associated with immune function. Total hemocyte frequencies, acid phosphatase activities, and superoxide production were all found to increase within 48 h of exposing oysters to magnesium chloride. In contrast, the phenoloxidase activities of oysters exposed to magnesium chloride declined significantly relative controls. All of these responses were relatively short term (96 h), again indicating an acute stress response.

Starvation also has a modulatory effect on Sydney rock oysters. Butt et al. (2007) demonstrated that the frequency of hemocytes and phenoloxidase activity in oyster hemolymph decreased by up to 25% in oysters whose diet had been halved relative to fully fed controls. Superoxide and peroxidase production also decreased significantly when oysters were starved (no food) for 2–4 weeks. All of these parameters returned to normal when starved oysters were fed. The recovery of phenoloxidase activities over-compensated during the recovery (full feeding) phase of the experiment to the extent that phenoloxidase activities post-recovery were substantially higher than those before starvation.

These data suggest that a range of well-defined stressors can affect the Sydney rock oyster immune system and may be associated with disease susceptibility. However, in some cases, the nature of the environmental perturbation associated with immune suppression and disease remains unknown. Butt and Raftos (2007) investigated a QX disease outbreak during 2005 in the Hawkesbury River, Sydney, in an effort to identify environmental variables associated with the disease in that river system. As in the Georges River, they found that phenoloxidase (and antimicrobial) activity was significantly inhibited during a key period of *M. sydneyi* infectivity (January–March 2005) and that phenoloxidase inhibition was strictly correlated with the intensity of *M. sydneyi* infection in oysters. The data indicated that some transient environmental stressor may have affected phenoloxidase activity during the critical infection window, increasing susceptibility of oysters to disease. However, the simultaneous analysis of a broad range of environmental variables (salinity, temperature, pH, algal density, chlorophyll a concentration, and dissolved oxygen) failed to identify any single factor that was associated with decreased phenoloxidase activity or disease intensity.

## Immunosuppression and disease susceptibility may be associated with programmed cell death

Even though there is strong evidence for a link between environmental stress and disease susceptibility, information is only now emerging to indicate a mechanistic basis for this association. A number of studies have shown that environmental stressors affect oyster hemocytes, which are the main mediators of immune responses. For instance, Kuchel et al. (2010b), Kuchel and Raftos (2011) investigated the effects of mechanical agitation, hypo-saline conditions, and exposure to the air on the hemocytes of Akoya pearl oysters (*Pinctada imbricata*). They found that both phagocytosis and phenoloxidase activity decreased significantly when oysters were exposed to all three stressors. Transient decreases were also evident in total hemocyte counts after mechanical stress and exposure to air, while significant increases in total hemocyte counts occurred after exposure to low salinity. Most significantly, the frequency of different hemocyte sub-populations in the hemolymph of *P. imbricata* was significantly altered when oysters were subjected hypo-saline conditions.

Lacoste et al. (2001a,b,c, 2002) began to uncover the mechanistic basis of these cellular responses to stress by demonstrating clear links between adrenergic stress responses, the suppression of cell-mediated immune responses, and disease susceptibility in Pacific oysters. This early work was then taken forward by Aladaileh et al. (2008a,b) and Kuchel and Raftos (2011). Aladaileh et al. (2008a) showed that noradrenaline secretion in Sydney rock oysters was stimulated by altered salinity, extremes of temperature, and physical agitation. This suggested that environmental factors that are commonly associated with oyster farming lead to adrenergic stress responses. The same study demonstrated that injecting noradrenaline into *S. glomerata* inhibits the phenoloxidase activities of both whole hemolymph and serum. It also decreases the frequency of hemocytes in hemolymph, alters differential hemocyte frequencies (including the frequency of phenoloxidase-positive cells), and inhibits phagocytic activity. Additional *in vitro* studies showed that the production of reactive oxygen intermediates, such as superoxide and peroxide, by hemocytes increased in the presence of noradrenaline.

**Figure 10:**
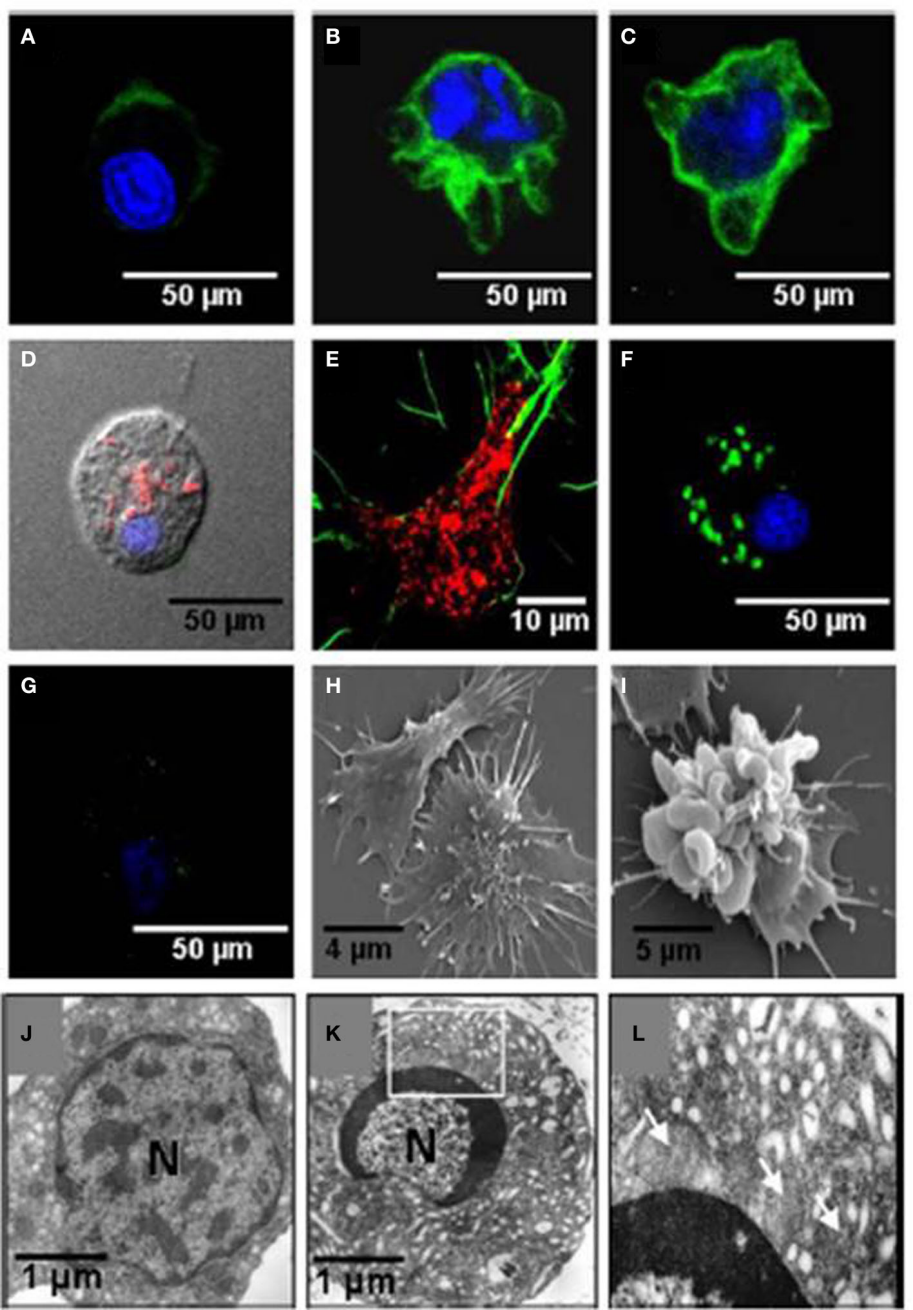
**Effects of noradrenaline on hemocytes morphology**. Hemocytes were stained for mitochondrial membrane potential (MitoTracker, red), F-actin (phalloidin-Alexa Fluor 488, green), and nuclear DNA (TO-PRO-3, blue). **(A–C)** Show stained hemocytes after different periods of noradrenaline treatment (10, 20, and 30 min, respectively). **(D,E)** are untreated hemocytes. **(F,G)** Show noradrenaline-untreated and treated hemocytes, respectively, stained for mitochondrial membrane potential with DiOC6(3) (green) and nuclear DNA with TO-PRO-3 (blue). **(H,I)** Show scanning electron micrographs of untreated hemocytes and noradrenaline-treated hemocytes, respectively. **(J,K)** Show transmission electron micrographs of untreated hemocytes, and noradrenaline-treated hemocytes, respectively. The box in **(K)** is enlarged in **(L)**. Mitochondria are shown by arrows. (Aladaileh et al., 2008b).

The effects of noradrenaline on the function of the Sydney rock oyster immune system were linked to changes in the composition of the defensive hemocyte population by Aladaileh et al. (2008b). Noradrenaline was shown to induce some of the typical features of programmed cell death (apoptosis) in *S. glomerata* hemocytes. These features included the loss of mitochondrial membrane potential, DNA fragmentation, and plasma membrane “blebbing.” Restructuring of the F-actin cytoskeleton was associated with these changes, which could explain why hemocyte adhesion and pseudopodia formation by hemocytes were inhibited by noradrenaline (Figure 10).

Similar observations have been made in the Akoya pearl oyster (*P. imbricata*) (Kuchel and Raftos, 2011; Kuchel et al., 2011). They found that treating *P. imbricata* hemocytes *in vitro* with noradrenaline resulted in enhanced DNA fragmentation relative to controls. Annexin V-FITC staining, a marker of early apoptotic events, and hemocyte adhesion were also significantly affected by exposure to noradrenaline. In addition, morphological and ultrastructural alterations that are typical of apoptosis were identified in noradrenaline treated hemocytes using transmission and scanning electron microscopy. These changes included chromatin and cytoplasmic condensation, the formation of apoptotic bodies, vacuolization, and blebbing. Polymerization of F-actin was also observed around the periphery of the cytoplasm.

All of these data support a model in which the apoptotic cell death caused by hormonal responses to environmental stress cause a depletion of critical hemocytes populations in oysters, resulting in immunosuppression and disease susceptibility.

## Conclusions and future directions

Infectious diseases are the main factors that limit the production of food and other products by aquaculture industries worldwide. Substantial evidence suggests that disease susceptibility in at least some aquaculture species, notably *S. glomerata*, is closely linked to the immunosuppressive effects of environmental stress. These effects seem to be mediated by hormonal stress responses that result in the apoptotic death of crucial hemocytes populations involved in immunological defense. Our next challenge is to understand the subcellular processes that result in apoptosis, and how these may be controlled to develop disease resistant populations for aquaculture. We are currently investigating whether environmental stress leads to increased metabolic activity with the consequent production of harmful ROS within oyster hemocytes that damages the cytoskeleton and initiates apoptosis. At a broader level, aquaculture industries worldwide need to start integrating our growing understanding of the cellular and genetic basis of disease resistance into effective management practices, such as marker assisted selection for disease resistance.

### Conflict of interest statement

The authors declare that the research was conducted in the absence of any commercial or financial relationships that could be construed as a potential conflict of interest.
